# Flexural Ductility and Strength in Hybrid FRP–Steel RC Beams

**DOI:** 10.3390/ma19132904

**Published:** 2026-07-06

**Authors:** Yanan Wu, Bo Chen, Sergio M. R. Lopes, Adelino V. Lopes, Yi Dong, Tiejiong Lou

**Affiliations:** 1 Sanya Science and Education Innovation Park, Wuhan University of Technology, Sanya 572000, China; 335254@whut.edu.cn (Y.W.); whutchenbo@gmail.com (B.C.); 2 School of Civil Engineering and Architecture, Wuhan University of Technology, Wuhan 430070, China; whutdongy@163.com; 3 CEMMPRE, ARISE, Faculty of Sciences and Technology, University of Coimbra, 3030-788 Coimbra, Portugal; sergio@dec.uc.pt; 4 INESC Coimbra, Department of Civil Engineering, University of Coimbra, 3030-788 Coimbra, Portugal; avlopes@dec.uc.pt; 5 PowerChina Jiangxi Electric Power Engineering Co., Ltd., Nanchang 330096, China

**Keywords:** hybrid RC beam, flexural strength, ductility index, finite element analysis

## Abstract

This study investigates hybrid fiber-reinforced polymer (FRP)–steel-reinforced concrete (RC) beams by using three-dimensional finite element models. The research systematically analyzes the influence of key parameters, including FRP type, FRP bar ratio (*ρ_f_*), the ratio of FRP to total reinforcement (*ρ_f_*/*ρ_t_*), and concrete strength. The load–deflection response of the hybrid RC beams is analyzed in detail. The results show that the investigated parameters have a relatively limited influence on the cracking moment, but significantly affect both the yield and ultimate moments. When *ρ_f_*/*ρ_t_* increases from 0 to 0.75, the yield moment decreases by up to 44.34%. When *ρ_f_* increases from 0.55% to 0.88%, the yield moment increases by 50.63%. Meanwhile, increasing the concrete strength significantly enhances the ultimate moment, with a maximum increase of 38.46%. In addition, an energy ductility index is adopted to quantitatively evaluate the structural ductility. The results indicate that the energy ductility index is consistently lower than the conventional ductility index. Finally, to improve the accuracy of theoretical predictions, a semi-empirical simplified formula is proposed for estimating the FRP bar stress at the ultimate state of hybrid beams. The verification results show that the proposed prediction method agrees well with the experimental data, demonstrating that the simplified formula has good applicability and reliability within the parameter range investigated in this study.

## 1. Introduction

Reinforced concrete (RC) structures—one of the conventional forms of modern civil engineering—are widely used in engineering construction because of their structural reliability, cost-effectiveness, and mature construction techniques. However, traditional steel bars are susceptible to corrosion during their service life [1,2]. In addition, RC structures have relatively high self-weight and substantial maintenance requirements, which together reduce durability and shorten service life [3,4]. To address these challenges, fiber-reinforced polymer (FRP) composites have gained increasing attention in the engineering field due to their low density, high strength, excellent resistance to environmental degradation, and strong fatigue durability. Various FRP systems—aramid (AFRP), basalt (BFRP), carbon (CFRP), and glass (GFRP)—have demonstrated substantial potential in mitigating the deterioration commonly associated with steel corrosion, thereby significantly enhancing structural durability and extending service life expectancy [5,6,7,8]. For instance, some countries have adopted FRP reinforcement instead of conventional steel bars in offshore bridge construction, which greatly reduces maintenance costs and extends structural service life. As a result, its overall life-cycle economic efficiency is significantly better than that of RC structures that require frequent maintenance and renovation [9].

Despite possessing multiple superior properties, FRP materials demonstrate an elastic modulus typically inferior to that of conventional steel bars. This mechanical limitation consequently results in pronounced deflection deformation and extensive crack propagation in FRP RC beams subsequent to tensile cracking [10]. More critically, FRP bars exhibit brittle failure mechanisms at ultimate limit states, lacking the yield plateau and plastic deformation capacity inherent to steel bars [11]. This deficit in ductility presents notable design challenges and raises safety concerns for structural applications. To overcome the inherent limitations of single-material reinforcement systems, an innovative hybrid reinforcement approach has been developed, wherein conventional steel bars are combined with FRP bars within the same structural element. This hybrid strategy is intended to capitalize on the complementary properties of both materials, leveraging the ductility and energy dissipation capacity of steel bars together with the high tensile strength and corrosion resistance of FRP bars. The adoption of such hybrid reinforcement has emerged as a promising research direction, with significant potential for enhancing structural durability, improving ductility performance, and optimizing serviceability, thereby contributing to the long-term structural integrity.

In recent years, the mechanical behavior and design methodology of hybrid FRP–steel RC beams have constituted a prominent research focus within structural engineering. Extant literature indicates that such hybrid reinforcement configurations effectively mitigate the inadequate stiffness and diminished ductility characteristic of FRP RC beams [12,13,14]. Kara et al. [15] developed a comprehensive numerical methodology for the prediction of curvature distribution, deflection response, and ultimate capacity, thereby providing a theoretical basis for optimizing reinforcement configurations. Experimental studies have confirmed that hybrid FRP–steel RC beams display deflection characteristics and average crack spacing intermediate between those of pure FRP beams and conventional RC beams [16]. This hybrid configuration effectively inhibits concrete crack propagation, reducing maximum crack widths by more than 50% [17]. Moreover, the inclusion of FRP bars not only reduces crack width but also enhances strength utilization efficiency by approximately 60% [18]. For equivalent reinforcement ratios, hybrid beams can achieve ultimate moment capacities ranging from 91% to 97% of those observed in conventional RC beams [19]. The ratio of FRP bar area to steel bar area (*A_f_*/*A_s_*) has emerged as a critical parameter governing the flexural behavior of hybrid beams. Recent studies have proposed and refined energy ductility indices and established *A_f_*/*A_s_* limits [20,21], indicating that within an optimal reinforcement range (*A_f_*/*A_s_* = 1.0–2.5), enhanced stiffness and load-carrying capacity can be achieved while maintaining satisfactory ductility. Further investigations reveal that increasing *A_s_*/*A_f_* results in hybrid beams that outperform conventional RC beams [22], with deflection capacities reaching 1.15–2.1 times those of RC beams [23]. Although flexural capacity can be improved by 15–45%, this enhancement is often accompanied by a reduction in ductility [24]. Therefore, the judicious allocation of steel and FRP bars is essential to compensate for the lower elastic modulus of FRP bars and to effectively enhance post-yield stiffness and flexural strength [25,26]. Furthermore, key factors including the FRP type, concrete compressive strength, and effective reinforcement ratio pronouncedly influence the ultimate flexural resistance, ductility, and deflection behavior of hybrid beams, prompting continuous refinement of relevant design methodologies [27,28,29].

Current design codes, such as CSA S806-12 [30], ACI 440.1R-15 [31], CEB-FIP 2007 [32], and GB 50608-2020 [33], have been primarily developed for FRP RC elements rather than hybrid reinforcement systems. These standards typically adopt the plane section assumption and strain compatibility principles, employing equivalent rectangular stress block methods to evaluate compressive stresses in the concrete and calculating tensile reinforcement forces based on strain distributions, cross-sectional areas, and material properties. Although these approaches provide a rational basis for structural design, they are mainly applicable to members reinforced with a single type of tensile reinforcement. In hybrid FRP–steel RC beams, the marked difference in elastic modulus between steel and FRP bars results in a more complex stress distribution across the cracked section. Therefore, direct application of existing code-based methods usually requires a tedious iterative procedure to determine the neutral axis depth and the corresponding stress state of each reinforcement component. As a result, current design codes provide limited guidance for hybrid members and often fail to achieve an appropriate balance between computational simplicity and accurate representation of the composite action between different reinforcement materials [34,35]. Therefore, further investigation into the stress mechanism of FRP bars at the ultimate limit state of hybrid beams is required. A simplified yet accurate ultimate stress model should be established to improve the calculation of ultimate flexural capacity and provide a theoretical basis for rapid preliminary strength estimation in engineering design.

Building upon the aforementioned research context, this study presents a systematic parametric study on simply supported hybrid FRP–steel RC beams. First, within a unified finite element (FE) framework and under clearly defined failure modes, the effects of key parameters, including FRP type, FRP bar ratio (*ρ_f_*), the ratio of FRP to total reinforcement (*ρ_f_*/*ρ_t_*), and concrete compressive strength, on the flexural capacity, deformation behavior, and ductility of hybrid beams are isolated and quantitatively evaluated. Second, the ductility of hybrid beams is quantitatively assessed by introducing an energy ductility index, which demonstrates a clear advantage in reflecting the actual energy dissipation capacity of the structure. Finally, based on reliable FE parametric analysis results and a critical evaluation of existing analytical models, an improved prediction method is proposed to estimate the ultimate stress of FRP bars and the flexural strength of hybrid beams. The proposed simplified model is extensively validated against a comprehensive experimental database, demonstrating its accuracy and reliability.

## 2. Numerical Procedure

### 2.1. Determination of Damage Patterns

In hybrid RC members, both FRP bars and steel bars collaboratively contribute to the transfer and resistance of external loads. However, pronounced differences in elastic modulus and ultimate strength between FRP and steel bars typically prevent the simultaneous onset of yielding or ultimate strength. The progressive interaction between steel and FRP bars under loading induces dynamic changes in stress transfer mechanisms and deformation compatibility, directly influencing the load-carrying behavior and determining the failure mode of the member. As a result, hybrid members can exhibit a wide spectrum of ultimate bearing capacities and distinct failure modes. To facilitate the analysis and design of such hybrid reinforcement systems, Pang et al. [20] introduced two nominal reinforcement indices—the effective reinforcement stiffness (*ρ_sf,s_*) and the mechanical reinforcement index (*ρ_sf,f_*)—to characterize the reinforcement properties of hybrid FRP–steel RC components based on equivalent elastic modulus and strength, respectively. The specific formulations are as follows:

(1) $$\rho_{sf,s}=\rho_{s}+\frac{E_{f}}{E_{s}}\rho_{f}$$

(2) $$\rho_{sf,f}=\rho_{f}+\frac{f_{y}}{f_{rup}}\rho_{s}$$

where *ρ_s_* and *ρ_f_* denote the effective reinforcement ratios of steel and FRP bars, respectively. *E_f_* and *E_s_* represent the elastic moduli of FRP and steel bars, respectively; *f_rup_* is the ultimate tensile strength of the FRP bars; and *f_y_* corresponds to the yield strength of the steel bars.

Given that the yield strain of steel bars is significantly lower than the ultimate strain of FRP bars, steel bars typically yield prior to FRP rupture during flexural loading, while FRP bars fail at much higher strain levels. Based on the preceding analysis, failure of hybrid structures at ultimate limit state can be classified into two primary categories: (1) compression failure, characterized by concrete crushing when it reaches its ultimate compressive strain; and (2) tension failure, manifested by FRP bar rupture when it attains its ultimate tensile strength. Owing to the comparatively lower yield strength of steel bars, in both failure scenarios, steel yielding typically precedes FRP rupture. The first balanced reinforcement ratio (*ρ_s_*_,_*_b_*) corresponds to a scenario in which steel yielding occurs simultaneously with concrete crushing, while the FRP bars remain unfractured. In contrast, the second balanced reinforcement ratio (*ρ_f_*_,_*_b_*) represents simultaneous occurrences in concrete crushing and FRP rupturing. Due to the relatively lower elastic modulus and higher ultimate tensile strength of FRP bars compared to steel bars, the *ρ_f_*_,_*_b_* is typically much smaller than that of *ρ_s_*_,_*_b_*, as defined by the following expression:

(3) $$\rho_{s,b}=0.85\beta_{1}\frac{f_{c}^{\prime }}{f_{y}}\frac{E_{s}\varepsilon_{cu}}{f_{y}+E_{s}\varepsilon_{cu}}$$

(4) $$\rho_{f,b}=0.85\beta_{1}\frac{f_{c}^{\prime }}{f_{rup}}\frac{E_{f}\varepsilon_{cu}}{f_{rup}+E_{f}\varepsilon_{cu}}$$

where $f_{c}^{\prime }$ denotes the compressive strength of a concrete cylinder; *ε_cu_* represents the ultimate compressive strain of concrete; and *β*_1_ is defined by $\beta_{1}=0.85-\left[0.05(f_{c}^{\prime }-28)/7\right]\geq 0.65$.

**Figure 1:**
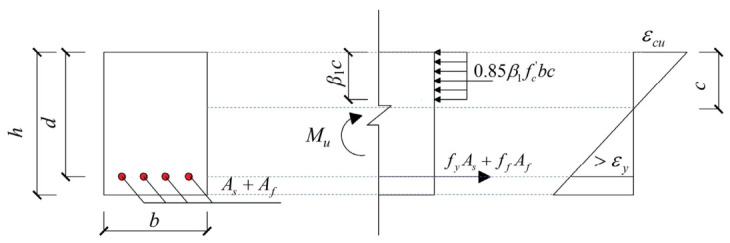
Strain and stress distribution at the ultimate stage.

When *ρ_sf,s_* exceeds *ρ_s_*_,_*_b_*, the *ρ_s_* surpasses the critical threshold without achieving yielding, resulting in excessive stress concentrations in the concrete compressive zone. This invariably precipitates concrete crushing as the governing failure mode—a scenario considered least desirable due to significantly diminished structural ductility and compromised safety. In contrast, when *ρ_sf_*_,_*_s_* ≤ *ρ_s_*_,_*_b_* and *ρ_sf_*_,_*_f_* ≥ *ρ_f_*_,_*_b_*, both steel yielding and concrete crushing occur prior to the attainment of ultimate capacity, allowing the FRP bars to fully realize their load-bearing contribution. Under these conditions, the hybrid RC beam effectively integrates the superior ductility of steel with the enhanced load-carrying capacity provided by FRP, thereby achieving an optimal failure mode. Conversely, when *ρ_sf_*_,*f*_ < *ρ_f_*_,*b*_, the *ρ_f_* falls below the critical threshold, rendering the beam prone to FRP tensile rupture before concrete crushing—resulting in a brittle failure mechanism that severely compromises structural safety. To address these safety concerns while maintaining structural efficiency, this study adopts the second failure mode as the primary design criterion. Based on this framework, an in-depth performance examination on hybrid beams is performed. Figure 1 illustrates the strain and stress distributions of hybrid beam under this failure mechanism. Here, *c* is the distance from the compressive ultimate fiber to the neutral axis under equilibrium strain conditions, *f_f_* is the tensile stress of the FRP bars, and *ε_y_* is the yield strain of the steel bars. *A_s_* and *A_f_* denote the cross-sectional areas of the steel bars and FRP bars, respectively; *b* and *h* represent the cross-sectional width and height, respectively; *d* is the distance from the compression limit fiber to the centroid of the bars.

### 2.2. Numerical Model

For compressive behavior of concrete, the constitutive model recommended by the European code [36] was employed in the numerical analysis. For tensile behavior of concrete, a bilinear constitutive model was employed, comprising an initial linear elastic phase preceding cracking and a subsequent linear hardening response post-cracking [37]. The constitutive behavior of steel bars was represented using a bilinear elastic–plastic model [38], while for the FRP bars, an ideal elastic–fracture model was utilized [3,6], wherein the material exhibits linear–elastic behavior up to its ultimate strain, beyond which brittle failure occurs. The concrete was modeled using the concrete damaged plasticity (CDP) model, with the key parameters defined as follows: dilation angle of 36°, flow potential eccentricity of 0.1, biaxial-to-uniaxial compressive strength ratio *f_b_*_0_*/f_c_*_0_ of 1.16, ratio of the second stress invariant on the tensile and compressive meridians K of 0.6667, and viscosity parameter of 0.001.

**Figure 2:**
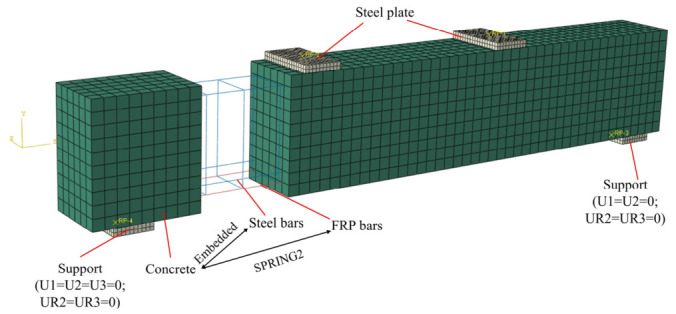
Details of FE model.

**Figure 3:**
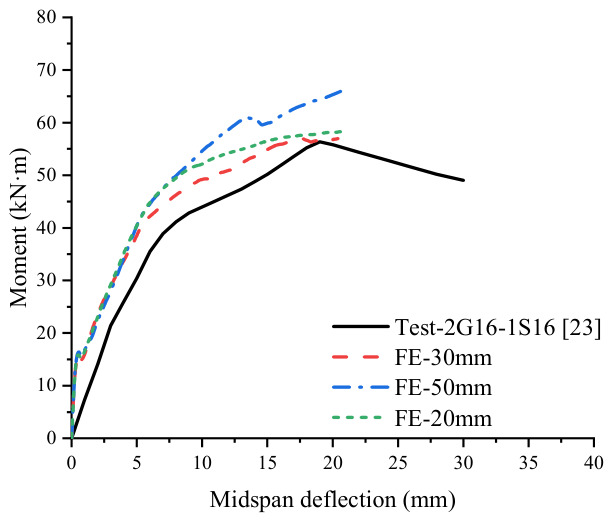
Analysis of mesh size sensitivity [23].

FE models were developed using ABAQUS 2022. To accurately capture the flexural response of the beams, the concrete was modeled using three-dimensional (3D) solid elements, while the internal steel/FRP bars were simulated using 2-node 3D truss elements, as depicted in Figure 2. A mesh sensitivity analysis was conducted using different mesh sizes of 20, 30, and 50 mm, as shown in Figure 3. Based on the comparison, a global mesh size of 30 mm was selected to balance computational accuracy and solution efficiency. Regarding the interfacial behavior, perfect bonding was assumed between the steel bars and the concrete; thus, the steel bars were directly embedded within the concrete elements. Conversely, because the bond–slip of FRP bars significantly influences the global flexural ductility of the hybrid beams [39], nonlinear spring elements were introduced at the FRP–concrete interface to explicitly simulate this interaction. Specifically, the interfacial bond–slip law was described using a modified BPE constitutive model [40], where the peak slip was set to 1.23 mm and the maximum bond stress was set to 11.6 MPa. Furthermore, vertical loads were applied to the model using displacement control.

### 2.3. Experimental Verification

(1) Verification 1

**Figure 4:**
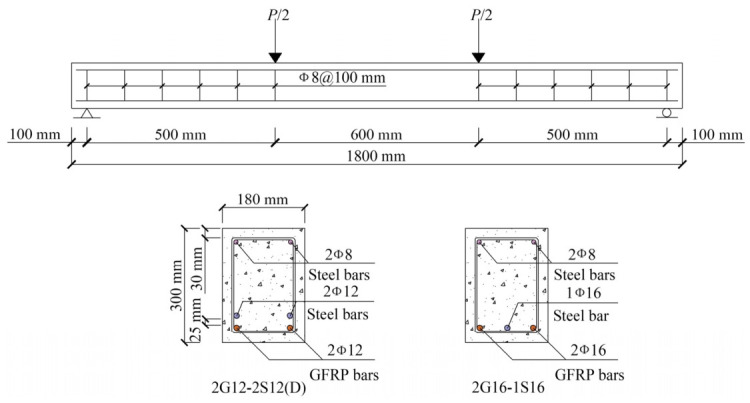
Details of Ruan beams [23].

Numerical simulations were performed on the hybrid GFRP–steel RC beams experimentally tested by Ruan et al. [23], and the predictions were validated against the experimental responses. The material properties assigned in the FE model were based on the measured experimental values. The 28-day concrete cube compressive strength (*f_cu_*) was 37.9 MPa. For the internal reinforcement, 8 mm diameter HPB300 grade steel bars were utilized for both the shear stirrups and the upper longitudinal steel bars. The bottom longitudinal tensile reinforcement comprised both steel and GFRP bars. The steel reinforcement included 12 mm (*f_y_* = 631 MPa) and 16 mm (*f_y_* = 643 MPa) diameter bars, both having an *E_s_* of 200 GPa. The GFRP reinforcement included 12 mm (*f_rup_* = 868.22 MPa, *E_f_* = 40.06 GPa) and 16 mm (*f_rup_* = 958.20 MPa, *E_f_* = 45.69 GPa) diameter bars. Detailed cross-sectional dimensions and reinforcement layouts of the specimens are illustrated in Figure 4.

**Figure 5:**
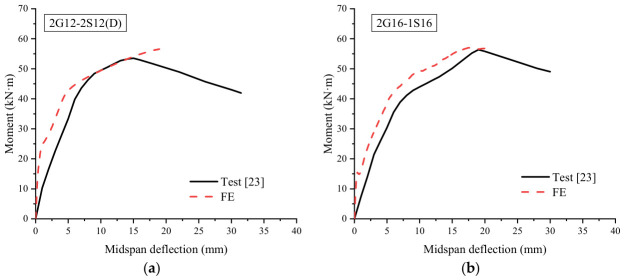
Moment-deflection curves [23]: (**a**) 2G12-2S12(D); (**b**) 2G16-1S16.

The FE analysis indicates concrete crushing failure, which is highly consistent with the experimental observations. Figure 5 compares the numerically and experimentally obtained moment–deflection curves. It can be observed that, before yielding of the tensile steel bars, the calculated initial stiffness is slightly higher than the experimental value due to the idealized assumptions adopted in the FE model. However, as the tensile steel bars enter the yielding stage, the stiffness degradation trend predicted by the model gradually agrees with the experimental curve.

(2) Verification 2

**Figure 6:**
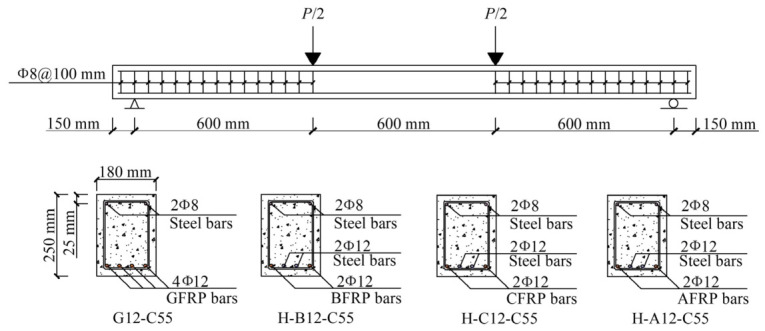
Details of Wei beams reprinted/adapted from Ref. [28].

This study further selected four specimens from the flexural tests conducted by Wei et al. [28], namely G12-C55, H-B12-C55, H-C12-C55, and H-A12-C55, reinforced with AFRP, CFRP, GFRP, and BFRP bars, respectively. The detailed specimen dimensions are shown in Figure 6. The material properties of the specimens were as follows: *f_cu_* = 70.31 MPa, *f_y_* = 521.2 MPa, *f_u_* = 642 MPa, and *E_s_* = 206.05 GPa. The elastic moduli of the AFRP, BFRP, CFRP, and GFRP bars were 50.1 GPa, 55.6 GPa, 124.2 GPa, and 45.04 GPa, respectively, while their corresponding ultimate tensile strengths were 1306.2 MPa, 912.66 MPa, 2102.1 MPa, and 910.56 MPa.

**Figure 7:**
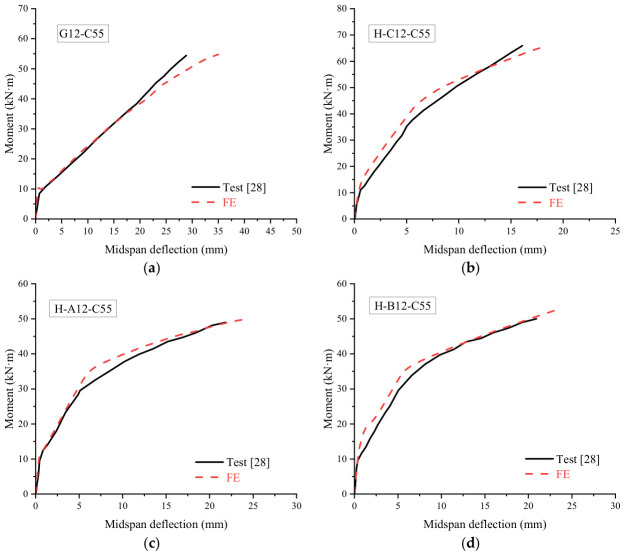
Moment-deflection curves [28]: (**a**) G12-C55; (**b**) H-C12-C55; (**c**) H-A12-C55; (**d**) H-B12-C55.

Figure 7 compares the measured and FE-predicted midspan moment–deflection responses of the four specimens. The FE results indicate that, when the specimens reached their ultimate load capacity, the bottom tensile steel bars had fully yielded, whereas none of the FRP bars reached their ultimate tensile strength or ruptured. The final failure was governed by crushing of the concrete in the midspan compression zone, which is consistent with the experimental failure mode reported by Wei et al. [28]. As shown in Figure 5, the FE model accurately captures the key flexural response characteristics of the specimens reinforced with AFRP, CFRP, GFRP, and BFRP bars, similar to the observations in Verification 1. By introducing this independent experimental dataset covering four different FRP material systems for further validation, the applicability of the developed FE model is further confirmed, supporting its reliable use in the subsequent parametric analysis.

### 2.4. Numerical Specimens

**Figure 8:**
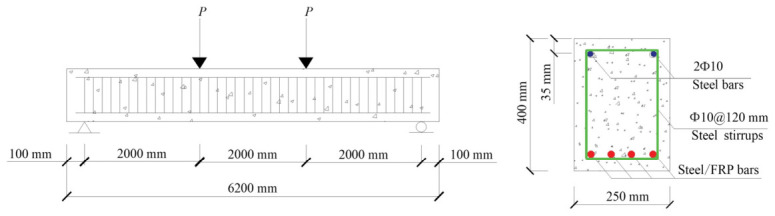
Schematic diagram of hybrid FRP–steel RC beam.

A conventional RC beam (S-0-30 beam) was modeled as the control specimen. The overall geometry, boundary conditions, and reinforcement details of the numerical models are illustrated in Figure 8. In these models, steel stirrups were represented by reinforcement with an elastic modulus of 190 GPa and a yield strength of 300 MPa, arranged at uniform intervals of 120 mm. The FRP longitudinal reinforcement types included AFRP, BFRP, CFRP, and GFRP bars, with respective elastic moduli of 80 GPa, 55 GPa, 133 GPa, and 40 GPa, and corresponding ultimate tensile strains of 2.5%, 1.6%, 1.28%, and 2%. The primary parameters of all beams are listed in Table 1. According to FE simulations, except for specimen A-1.0-30, failure for all specimens has resulted from crushing of concrete following yielding of steel bars, while FRP bars have not reached their ultimate tensile strength at failure.

## 3. Flexural Ductility

### 3.1. Load—Deflection Behavior

**Figure 9:**
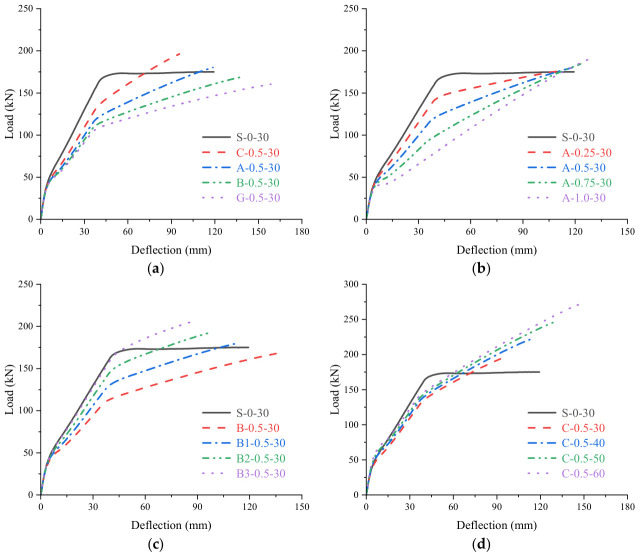
Load–deflection curves: (**a**) Influence of FRP type; (**b**) Influence of *ρ_f_*/*ρ_t_*; (**c**) Influence of *ρ_f_*; (**d**) Influence of concrete strength.

Figure 9 illustrates the evolution of the mid-span load–deflection response for hybrid FRP–steel RC beams. For the A-1.0-30 beam, which is reinforced solely with AFRP bars, the curve remains linear elastic after concrete cracking, with no discernible yielding plateau, and exhibits a characteristic bilinear response. In contrast, the load–deflection curves of the other hybrid beams closely resemble that of the S-0-30 beam, exhibiting a distinct trilinear behavior. The transition points observed on these curves correspond to the cracking of concrete and the yielding of steel bars, effectively dividing the response into three stages: (i) pre-cracking of concrete, (ii) post-cracking of concrete, and (iii) post-yielding of steel bars. Notably, at comparable load levels, the S-0-30 beam demonstrates a more gradual increase in deflection.

Figure 9a demonstrates that the FRP type exerts a substantial influence on the performance of hybrid RC beams. Since this study focuses on beams governed by concrete crushing failure, the post-cracking stiffness evolution of the members and the stress contribution of the FRP reinforcement are mainly controlled by the elastic modulus of the FRP bars rather than their ultimate strain. Therefore, the following discussion primarily focuses on the influence of the elastic modulus. An increase in the elastic modulus of the FRP bars leads to a corresponding increase in the ultimate bearing capacity of the beams. Notably, the load–deflection curve for the C-0.5-30 beam, which is reinforced with CFRP bars, displays a considerably steeper slope compared to those of other beams, indicative of superior stiffness. The ultimate bearing capacities of the C-0.5-30 beam are 8.22%, 14.19%, and 18.34% higher than those of the A-0.5-30, B-0.5-30, and G-0.5-30 beams, respectively. However, the ultimate deflection of the C-0.5-30 beam is significantly lower, with reductions of 24.1%, 43.2%, and 65.8% relative to the aforementioned beams. This behavior is chiefly attributed to the markedly higher elastic modulus and ultimate tensile strength of CFRP bars relative to other FRP types, underscoring the pivotal role of FRP type in hybrid beams.

Figure 9b demonstrates that the effect of *ρ_f_*/*ρ_t_* becomes increasingly significant after concrete cracking. Both ultimate load and displacement increase as *ρ_f_*/*ρ_t_* rises, and at any given load, beams with a larger *ρ_f_*/*ρ_t_* exhibit more rapid deflection growth. For instance, increasing *ρ_f_*/*ρ_t_* from 0.25 to 1.0 results in an approximate 7.49% increase in ultimate load (from 176.7 kN to 189.93 kN) and a 13.35% increase in ultimate deflection (from 112.61 mm to 127.64 mm). Comparatively, Figure 9c shows that the influence of the *ρ_f_* on flexural performance is opposite to that of *ρ_f_*/*ρ_t_*. When the *ρ_f_* increases from 0.55% to 0.88%, the ultimate load increases by 22.68%, while the ultimate deflection decreases from 136.94 mm to 88.59 mm (a reduction of 35.31%). These findings suggest that while a higher reinforcement ratio enhances load-carrying capacity, it may concurrently suppress deflection capacity, highlighting the critical regulatory role of *ρ_f_* in the flexural behavior of hybrid beams.

Figure 9d demonstrates that concrete strength effects begin from the initial loading phase. As concrete strength increases, both initial stiffness and cracking load are elevated. Specifically, increasing the concrete strength from C30 to C60 results in a 54.04% increase in ultimate deflection (from 95.66 mm to 147.35 mm) and an approximate 38.46% increase in ultimate bearing capacity (from 196.41 kN to 271.94 kN). The results indicate that increasing concrete strength markedly enhances the overall flexural performance of hybrid beams.

### 3.2. Ductility Performance

#### 3.2.1. Conventional Ductility Index

The ductility index (*μ*_∆_) quantifies a member’s capacity to sustain deformation beyond first yielding. It is defined as the ratio of the deflection at the ultimate state (Δ*_u_*) to that corresponding to the yield moment (Δ*_y_*), as follows:

(5) $$\mu_{\Delta }=\frac{\Delta_{u}}{\Delta_{y}}$$

For specimens investigated herein, only the S-0-30 beam is suitable for evaluation using conventional ductility index, yielding a ductility index of 3.03. However, for hybrid FRP–steel RC beams, conventional ductility indices fail to accurately characterize true ductility performance. This shortcoming arises because, as *ρ_f_*/*ρ_t_* increases, the tensile steel reinforcement yields at an earlier stage, resulting in a notably lower yield deflection compared to conventional RC beams. Nevertheless, after steel yielding, the FRP bars remain within their linear elastic range and continue to carry tensile forces, producing a substantial increase in the ultimate deflection prior to concrete crushing. Consequently, continued reliance on the conventional ductility index may misleadingly suggest that hybrid RC beams possess significantly higher ductility than conventional beams, solely due to the earlier occurrence of yield deflection—an interpretation inconsistent with the actual stress distribution and post-yield behavior of such systems. Therefore, it is essential to establish a new ductility evaluation index that simultaneously captures the yielding characteristics of the steel bars and the sustained tensile contribution of the FRP bars, thereby enabling more accurate and objective assessments on ductility behavior of hybrid beams.

#### 3.2.2. Energy Ductility Index

**Figure 10:**
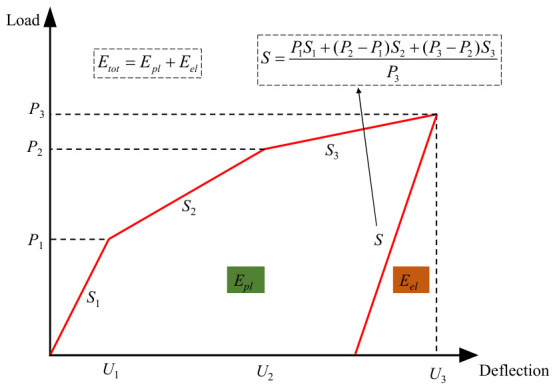
Definition of energy ductility index [28,41].

Naaman and Jeong [41] proposed an energy ductility index (*μ_e_*) for FRP RC beams (see Figure 10). This index evaluates plastic energy dissipation and ductility reserve by taking the ratio of the total absorbed energy to the elastic energy at yield, thus providing a comprehensive measure of post-cracking energy dissipation capacity. The *μ_e_* is defined as:

(6) $$\mu_{e}=\frac{1}{2}\left(\frac{E_{tot}}{E_{el}}+1\right)$$

where *E_tot_* is the total energy absorbed by the beam from initial loading up to failure; *E_pl_* represents the plastic energy dissipated prior to failure; and *E_el_* denotes the elastic energy released after failure.

**Figure 11:**
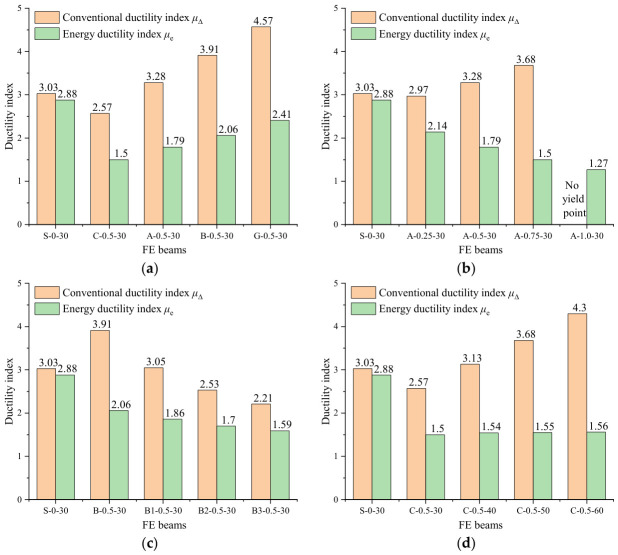
Effect of different parameters on ductility index: (**a**) Influence of FRP type; (**b**) Influence of *ρ_f_*/*ρ_t_*; (**c**) Influence of *ρ_f_*; (**d**) Influence of concrete strength.

Figure 11 systematically compares the results obtained from the conventional ductility index and the energy ductility index. Except for the S-0-30 beam, there are significant differences between the two ductility indicators for the other hybrid beams. This suggests that the conventional ductility index is insufficient for accurately characterizing the true ductility performance of these systems [27]. The FRP type has a pronounced effect on the energy ductility index, as demonstrated in Figure 11a. Specifically, as the elastic modulus of the FRP decreases, the energy ductility index increases. For instance, the energy ductility indices of hybrid GFRP–steel RC beams are 1.17, 1.35, and 1.61 times those of hybrid BFRP–steel, AFRP–steel, and CFRP–steel RC beams, respectively, indicating that using FRP with a higher elastic modulus yields a lower energy ductility index. Figure 11b illustrates the effect of *ρ_f_*/*ρ_t_* on the ductility index, showing that the energy ductility index decreases progressively as *ρ_f_*/*ρ_t_* increases, whereas the conventional ductility index exhibits the opposite trend. This divergence arises because increasing *ρ_f_*/*ρ_t_* causes the tensile steel bars to yield earlier, reducing the yield deflection and artificially inflating the conventional ductility index due to the “forward shift” of the yield point. Additionally, Figure 11c demonstrates that with increasing *ρ_f_*, the energy ductility index of hybrid RC beams exhibits a decreasing trend. Figure 11d indicates that concrete strength exerts a relatively limited influence, with only minor increases in the energy ductility index observed across different strength grades. Overall, the energy ductility index of hybrid RC beams is consistently lower than that of conventional RC beams, providing a more accurate representation of true ductility under varying parameters.

## 4. Flexural Strength

### 4.1. Cracking Moment

**Figure 12:**
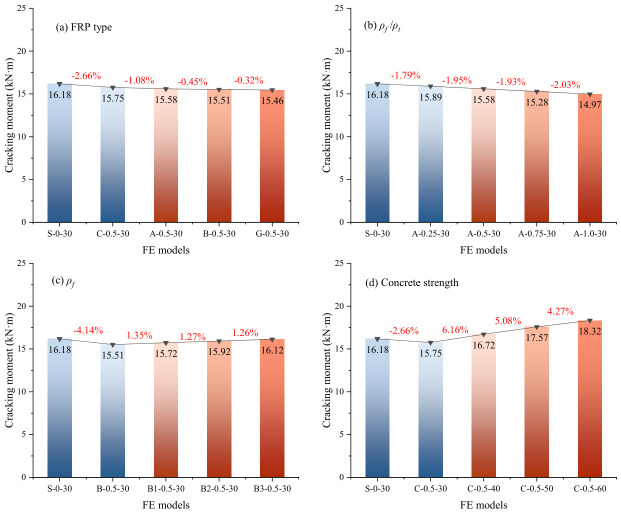
Effect of different parameters on cracking moment: (**a**) Influence of FRP type; (**b**) Influence of *ρ_f_*/*ρ_t_*; (**c**) Influence of *ρ_f_*; (**d**) Influence of concrete strength.

Figure 12 shows that the cracking moment of hybrid RC beams is influenced by several factors. Overall, the influence of these parameters on the cracking moment is relatively limited; within the parameter ranges considered, the maximum variation does not exceed 10%. However, each parameter exhibits distinct trends and mechanisms of action. As shown in Figure 12a, an increase in the elastic modulus of FRP bars elevates the cracking moment. The underlying mechanism is the increase in equivalent tensile stiffness, which reduces tensile strain in the pre-cracking stage, delays the attainment of the concrete tensile strength, and thus raises the initial cracking moment. Conversely, Figure 12b indicates that increasing the *ρ_f_*/*ρ_t_* reduces the cracking moment, owing primarily to the generally lower elastic modulus of FRP bars relative to steel bars. Figure 12c reveals that *ρ_f_* exerts only a minor effect on the cracking moment, with a moderate increase in *ρ_f_* providing a slight improvement in section tensile stiffness and a marginal rise in cracking moment. Finally, as depicted in Figure 12d, increasing concrete strength enhances the cracking moment, attributed to the greater tensile strength associated with high-strength concrete.

### 4.2. Yield Moment

**Figure 13:**
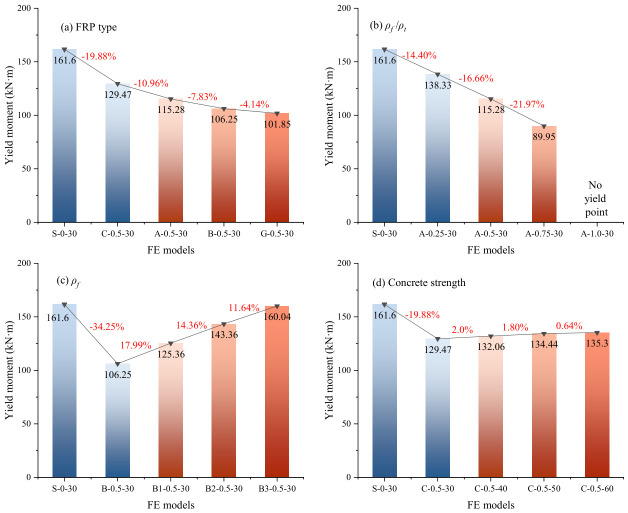
Effect of different parameters on yield moment: (**a**) Influence of FRP type; (**b**) Influence of *ρ_f_*/*ρ_t_*; (**c**) Influence of *ρ_f_*; (**d**) Influence of concrete strength.

Figure 13 systematically evaluates the influence of various parameters on the yield moment of hybrid beams. Notably, the A-1.0-30 beam, which is reinforced exclusively with AFRP bars, exhibits a linear elastic response after cracking up to the point of concrete crushing; as a result, it does not possess a conventional yield moment. Figure 13a indicates that the FRP type is a key factor influencing the yield moment of hybrid beams. In hybrid FRP–steel RC beams, those incorporating FRP bars with lower elastic moduli exhibit correspondingly reduced yield moment. Figure 13b demonstrates that *ρ_f_*/*ρ_t_* is a critical parameter, as the yield moment decreases by up to 44.34% when *ρ_f_*/*ρ_t_* increases from 0 to 0.75. Additionally, Figure 13c indicates that *ρ_f_* is also an important parameter; increasing *ρ_f_* from 0.55% to 0.88% results in a 50.63% increase in the yield moment. This enhancement is attributed to the improved flexural stiffness and reserve capacity provided by additional FRP bars, which delays the onset of steel yielding and consequently increases the yield moment. In contrast, Figure 13d shows that concrete strength exerts a negligible effect on the yield moment; even as concrete strength increases from 30 MPa to 60 MPa, the yield moment only increases from 129.47 kN·m to 135.3 kN·m. This limited impact is mainly because the yield moment is predominantly controlled by the stress–strain characteristics of the tensile reinforcement, while the concrete primarily contributes to the compressive zone. Thus, when the quantity or strength of the tensile reinforcement is sufficient, further increases in concrete strength have only a marginal influence on the yield behavior of the steel bars.

### 4.3. Ultimate Moment

Compared with conventional RC beams (e.g., S-0-30 beam), hybrid FRP–steel RC beams benefit from the continued tensile contribution of the FRP bars after steel yielding, leading to a pronounced increase in load-carrying capacity at the ultimate moment stage. For instance, in the S-0-30 beam, the yield moment is 161.6 kN·m and the ultimate moment is 174.96 kN·m, reflecting only a modest increase beyond yielding. In contrast, hybrid beams capitalize on the continued tensile capacity of the FRP bars, which remain linearly elastic beyond the yield point of steel. This leads to a more substantial enhancement in the section’s ultimate load capacity during the progression from yield to ultimate states.

**Figure 14:**
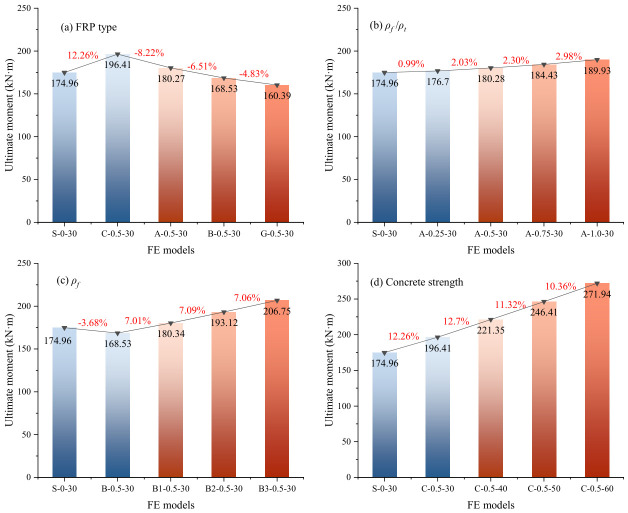
Effect of different parameters on ultimate moment: (**a**) Influence of FRP type; (**b**) Influence of *ρ_f_*/*ρ_t_*; (**c**) Influence of *ρ_f_*; (**d**) Influence of concrete strength.

As shown in Figure 14a, increasing the elastic modulus of the FRP bars significantly raises the ultimate moment. Once the steel yields, the FRP bars assume the primary tensile role; a higher elastic modulus enhances their tensile contribution and extends the yield-to-limit development interval associated with post-crack curvature, thereby increasing the ultimate moment. Furthermore, Figure 14b demonstrates that the ultimate moment exhibits a significant positive correlation with *ρ_f_*/*ρ_t_*. Specifically, as *ρ_f_*/*ρ_t_* increases from 0 to 0.25, 0.5, 0.75, and 1.0, the ultimate moment increases by 0.99%, 3.04%, 5.41%, and 8.56%, respectively, compared with the reference beam. Figure 14c demonstrates that increasing *ρ_f_* can also significantly enhance the ultimate moment. As *ρ_f_* increases, the post-yield tensile contribution of the FRP bars increases, facilitating a more favorable redistribution of internal forces within the section. This effect delays the attainment of either the FRP ultimate tensile strain or the concrete’s ultimate compressive strain in the compression zone, thereby improving the ultimate load-carrying capacity. Similarly, Figure 14d indicates that higher concrete strength grades result in improved ultimate moments, primarily due to the greater compressive capacity provided by the enhanced concrete, which postpones the onset of ultimate compressive strain.

### 4.4. Predictive Flexural Strength Models

Given that hybrid FRP–steel RC beams can reasonably be assumed to satisfy the plane section hypothesis in flexural failure analysis, a simplified calculation model recommended by ACI 440.1R-15 [31]—based on sectional force equilibrium and strain compatibility—can be adopted to determine the FRP stress (*f_f_*) and the corresponding ultimate flexural capacity (*M_u_*) of hybrid beams:

(7) $$f_{f}=\sqrt{\frac{1}{4}(\frac{A_{s}f_{y}}{A_{f}}+E_{f}\varepsilon_{cu}{)}^{2}+(0.85\frac{\beta_{1}f_{c}^{\prime }}{\rho_{f}}-\frac{A_{s}f_{y}}{A_{f}})E_{s}\varepsilon_{cu}}-\frac{1}{2}(\frac{A_{s}f_{y}}{A_{f}}+E_{f}\varepsilon_{cu})\leq f_{rup}$$

(8) $$M_{u}=(\rho_{s}f_{y}+\rho_{f}f_{f})(1-0.59\frac{\rho_{s}f_{y}+\rho_{f}f_{f}}{f_{c}^{\prime }})bd^{2}$$

The equation for calculating the FRP stress in hybrid beams provided in ACI 440.1R-15 [31], as shown in Equation (7), involves many parameters and requires considerable calculation effort, which limits its practicality and applicability in engineering design. To address these issues, Wei et al. [28] proposed a simplified formula for *f_f_* applicable to hybrid RC beams, which is expressed as follows:

(9) $$f_{f}=(371.48+6.19E_{f})(0.485+0.009f_{c}^{\prime })(-325.35\rho_{sf,s}+3.39)$$

Equation (9) simplifies Equation (7) by incorporating the influence of the comprehensive parameter *ρ_sf,s_*, thereby enhancing computational efficiency. However, this combined treatment reduces the ability to distinguish the individual contributions of *A_s_*/*A_f_* (or *A_f_*/*A_t_*) and *ρ_f_* to the stress distribution and failure mechanisms. Moreover, the simplified model currently lacks sufficient experimental validation and numerical simulation support, suggesting that its general applicability and reliability require further evaluation.

**Figure 15:**
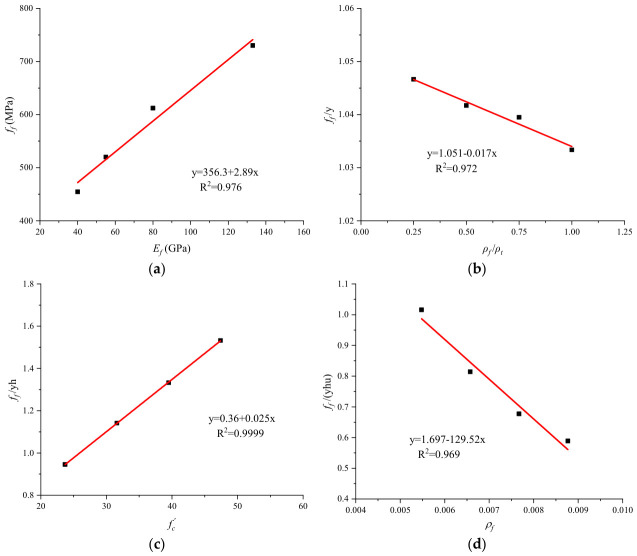
Fitted curves for different parameters: (**a**) *E_f_*; (**b**) *ρ_f_*/*ρ_t_*; (**c**) $f_{c}^{\prime }$; (**d**) *ρ_f_*.

Based on the above understanding, this study systematically incorporates the mechanisms of key parameters, including *E_f_*, *ρ_f_*/*ρ_t_*, $f_{c}^{\prime }$ and *ρ_f_*, through detailed FE parameter analyses, and subsequently proposes a predictive model for *f_f_* of hybrid RC beams at the ultimate limit state. Based on the fitted relationships between *f_f_* and the principal parameters illustrated in Figure 15, the following expression has been derived:

(10) $$f_{f}=y(E_{f})=356.3+2.89E_{f}$$

(11) $$\frac{f_{f}}{y}=h(\frac{\rho_{f}}{\rho_{t}})=1.051-0.017(\frac{\rho_{f}}{\rho_{t}})$$

(12) $$\frac{f_{f}}{\left(yh\right)}=u(f_{c}^{\prime })=0.36+0.025f_{c}^{\prime }$$

(13) $$\frac{f_{f}}{\left(yhu\right)}=w(\rho_{f})=1.697-129.52\rho_{f}$$

In summary, the simplified formula for the stress expression can be presented as follows:

(14) $$f_{f}=(356.3+2.89E_{f})(1.051-0.017\frac{\rho_{f}}{\rho_{t}})(0.36+0.025f_{c}^{\prime })(1.697-129.52\rho_{f})$$

### 4.5. Model Validation

**Figure 16:**
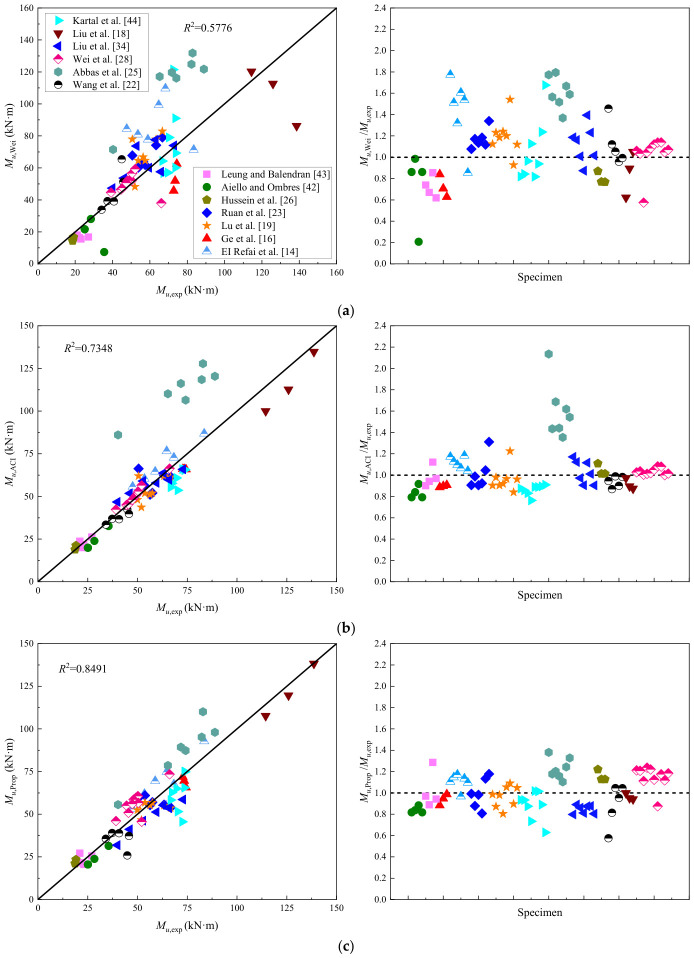
Correlation between the predictions of three simplified models and experimental results [14,16,18,19,22,23,25,26,28,34,42,43,44]: (**a**) Simplified model of Wei et al. [28]; (**b**) Simplified model of ACI 440.1R-15 [31]; (**c**) Simplified model of this paper.

To verify the applicability and robustness of the simplified formula proposed in this study, Table 2 summarizes 74 sets of hybrid RC beam test data from the existing literature. Three simplified prediction methods—ACI 440.1R-15 [31], the Wei et al. model [28], and the model proposed in this study—are compared and evaluated. Figure 16 further presents scatter plots and the deviation distribution of ultimate moment predictions by each method in comparison with experimental values. Analysis of the data in Figure 16 and Table 2 reveals that the Wei et al. model [28] exhibits significant systematic deviation and fluctuation, with an average theoretical-to-experimental ratio of 1.103 and a standard deviation of 0.313, indicating limited accuracy and stability. The ACI 440.1R-15 [31] outperforms the Wei et al. model [28], achieving a mean ratio of 1.035 and a standard deviation of 0.223, which demonstrates greater robustness and practicality for engineering applications. In comparison, the simplified prediction model proposed in this paper demonstrates clear advantages in predicting the ultimate moment of hybrid RC beams. The average theoretical-to-experimental ratio is 1.006, with a standard deviation of only 0.163, which is markedly better than those of the other models. These results highlight that the proposed formula not only achieves higher prediction accuracy but also exhibits superior stability, thereby providing a more reliable theoretical basis for the design and analysis of hybrid RC beams.

## 5. Conclusions

In this study, numerical models of 14 beams were established using FE software, consisting of 13 hybrid FRP–steel RC beams and one conventional RC beam. A systematic analysis was conducted on the failure modes, load-deformation and ductility performance, and flexural strength of the beams. Emphases were placed on the influence of key parameters, i.e., FRP type, *ρ_f_*/*ρ_t_*, concrete strength, and *ρ_f_*. Furthermore, based on the evaluation of existing simplified models, an improved theoretical model for predicting the flexural strength of hybrid beams was developed. Major research conclusions are summarized as follows:

- Parameter sensitivity analysis of the ultimate-state performance shows that increasing *E_f_* significantly improves the ultimate load of hybrid FRP–steel RC beams, while reducing the ultimate deflection. In particular, the hybrid CFRP–steel RC beam achieves an ultimate bearing capacity 8.22–18.34% higher than other hybrid FRP beams, and its deflection is reduced by 24.1–65.8%. As *ρ_f_*/*ρ_t_* increases, both the ultimate load and deflection increase synchronously, by 7.49% and 13.35%, respectively. Further increasing *ρ_f_* continues to enhance the bearing capacity, but may result in a maximum deflection reduction of 35.31%. Concurrently, improved concrete strength not only enhances ultimate load but also positively impacts structural ductility performance.
- The results calculated using the energy ductility index for hybrid FRP–steel RC beams are significantly lower than those obtained from conventional ductility index, providing a more accurate reflection of the actual ductility level of the hybrid reinforcement system. Parametric sensitivity analysis demonstrates that a reduction in *E_f_* significantly enhances the ductility index, whereas increases in *ρ_f_*/*ρ_t_* and *ρ_f_* result in a decrease in structural ductility. In contrast, variations in concrete strength have a relatively limited effect on the ductility index.
- Impacts of varying parameters on cracking moment in hybrid RC beams are relatively limited, with variations generally constrained within 10%. By contrast, the *E_f_*, *ρ_f_*/*ρ_t_*, and *ρ_f_* exert pronounced effects on the yield moment. Specifically, a lower Ef leads to a reduced beam yield moment. Furthermore, when *ρ_f_*/*ρ_t_* increases from 0 to 0.75, the maximum reduction in yield moment reaches 44.34%; increasing *ρ_f_* from 0.55% to 0.88% results in a corresponding increase in the yield moment of 50.63%. In comparison, the contribution of concrete strength is comparatively minor: increasing the concrete strength from 30 MPa to 60 MPa yields only a 4.5% increase in the yield moment. With respect to the ultimate moment, increasing any of the parameters considered here can substantially enhance the ultimate capacity of the beams.
- Predictions in flexural strength by the simplified model of Wei et al. [28] exhibit significant discrepancies when compared with the experimental values. In contrast, ACI 440.1R-15 [31] provides more accurate predictions than the model of Wei et al. [28]. Notably, the simplified model developed in this study exhibits the highest prediction accuracy, with a mean value for *M_u_*/*M_u_*_,exp_ of 1.006 and a standard deviation of only 0.163. This indicates that the proposed model has good applicability and stability.
- The simplified formulation presented in this study can be effectively used for preliminary estimation of bearing capacity within its applicable parameter range. However, for formal engineering design, it remains necessary to strictly comply with the relevant design specifications and to incorporate appropriate safety factors and construction requirements to ensure adequate structural safety margins.

## Figures and Tables

**Table 1:** Main parameters of numerical simulation beams.

Beam	Tensile Reinforcement Ratio (%)		*ρ_f_*/*ρ_t_*	*ρ_s_*_,_*_b_* (%)	*ρ_f_*_,_*_b_* (%)	*ρ_sf_*_,_*_s_* (%)	*ρ_sf_*_,_*_f_* (%)
	*ρ_s_*	*ρ_f_*					
S-0-30	1.10	-	-	2.60	-	-	1.10
A-0.25-30	0.82	0.28	0.25	2.60	0.13	0.93	0.47
A-0.5-30	0.55	0.55	0.50	2.60	0.13	0.77	0.68
A-0.75-30	0.28	0.82	0.75	2.60	0.13	0.60	0.89
A-1.0-30	-	1.10	1.00	-	0.13	-	-
B-0.5-30	0.55	0.55	0.50	2.60	0.43	0.70	0.85
B1-0.5-30	0.66	0.66	0.50	2.60	0.43	0.84	1.02
B2-0.5-30	0.77	0.77	0.50	2.60	0.43	0.98	1.19
B3-0.5-30	0.88	0.88	0.50	2.60	0.43	1.12	1.36
C-0.5-30	0.55	0.55	0.50	2.60	0.27	0.91	0.70
C-0.5-40	0.55	0.55	0.50	3.16	0.33	0.91	0.70
C-0.5-50	0.55	0.55	0.50	3.59	0.37	0.91	0.70
C-0.5-60	0.55	0.55	0.50	4.04	0.42	0.91	0.70
G-0.5-50	0.55	0.55	0.50	2.60	0.40	0.66	0.88

Note: Each letter in the table represents the bar type, “A”, “B”, “C”, and “G” refer to AFRP, BFRP, CFRP, and GFRP bars, respectively. For example, the designation “C-0.5-50” indicates a hybrid beam reinforced with CFRP and steel bars, where the *ρ_f_*/*ρ_t_*is 0.50, and *f_cu_* is 50 MPa.

**Table 2:** Comparison of experimental ultimate flexural strength with predictions from three simplified models for hybrid FRP–steel RC beams.

Reference	Specimen	*E_f_* (GPa)	*ρ_f_*/*ρ_t_*	*ρ_f_*	$f_{c}^{\prime }$ (MPa)	ACI440.1R-15 [31]		Proposed		Wei et al. [28]		*M_u_*_,exp_	*M_u_*/*M_u_*_,exp_		
						*f_f_* (MPa)	*M_u_*_,ACI_ (kN·m)	*f_f_* (MPa)	*M_u_*_,Prop_ (kN·m)	*f_f_* (MPa)	*M_u_*_,Wei_ (kN·m)		ACI [31]	Wei et al. [28]	Proposed
Aiello and Ombres [42]	A1	49.0	0.47	0.0034	36.1	777.16	19.90	827.03	20.55	912.10	21.65	25.14	0.791	0.861	0.817
	A2	50.1	0.61	0.0060	36.1	609.93	23.84	607.25	23.78	800.25	27.99	28.41	0.839	0.985	0.837
	A3	50.1	0.51	0.0090	36.1	391.61	32.57	352.81	31.39	-336.91	7.37	35.55	0.916	0.207	0.883
	C1	49.0	0.47	0.0034	36.1	777.16	19.90	827.03	20.55	912.10	21.65	25.14	0.791	0.861	0.817
Leung and Balendran [43]	L2	40.8	0.48	0.0059	27.08	383.36	19.97	475.62	21.51	174.07	16.41	22.20	0.900	0.739	0.969
	L5	40.8	0.58	0.0089	27.08	326.89	21.74	277.86	20.53	82.84	15.49	23.10	0.941	0.670	0.889
	H2	40.8	0.48	0.0059	46.36	510.47	23.65	696.77	27.11	216.96	18.01	21.10	1.121	0.854	1.285
	H5	40.8	0.58	0.0089	46.36	432.37	26.23	407.06	25.53	103.25	16.76	27.10	0.968	0.619	0.942
Ge et al. [16]	FS1	55.0	0.49	0.0057	28.1	549.85	65.81	548.18	65.70	497.11	62.50	74.4	0.885	0.840	0.883
	FS2	55.0	0.39	0.0047	28.1	548.84	65.91	619.27	69.52	287.56	51.92	73.5	0.897	0.706	0.946
	FS3	55.0	0.30	0.0038	28.1	547.65	66.02	690.49	71.85	78.00	45.63	72.8	0.907	0.627	0.987
EI Refai et al. [14]	2G12-1S10	50.0	0.74	0.0040	40.0	888.77	55.75	833.98	53.07	1503.72	84.41	47.62	1.171	1.773	1.115
	2G12-2S10	50.0	0.59	0.0040	40.0	802.54	60.31	836.05	61.91	1243.84	80.85	53.55	1.126	1.510	1.156
	2G12-2S12	50.0	0.50	0.0040	40.0	732.91	64.56	837.28	69.48	1015.16	77.69	58.94	1.095	1.318	1.179
	2G16-2S10	50.0	0.72	0.0072	40.0	607.71	72.74	544.02	67.52	1091.32	109.63	68.30	1.065	1.605	0.989
	2G16-2S12	50.0	0.64	0.0072	40.0	565.06	76.52	544.73	74.88	860.77	99.36	64.71	1.182	1.536	1.157
	2G16-2S16	50.0	0.50	0.0072	40.0	467.61	86.79	545.98	92.81	273.87	71.36	83.53	1.039	0.854	1.111
Ruan et al. [23]	2G12-2S12	40.06	0.50	0.0049	30.32	477.00	52.09	583.88	57.05	691.53	61.90	57.50	0.906	1.077	0.992
	2G16-2S12	45.69	0.64	0.0088	30.32	402.98	62.45	316.81	55.61	558.68	74.15	63.30	0.987	1.171	0.878
	2G12-1S16	40.06	0.53	0.0049	30.32	491.34	51.03	583.60	55.34	774.73	63.95	56.37	0.905	1.135	0.982
	2G16-1S16	45.69	0.67	0.0088	30.32	412.37	61.56	316.67	53.91	647.25	78.98	66.70	0.923	1.184	0.808
	2G12-2S12(D)	40.06	0.50	0.0049	30.32	477.00	56.15	583.88	61.00	564.86	60.14	53.79	1.044	1.118	1.134
	2G16-2S12(D)	45.69	0.64	0.0088	30.32	402.98	66.27	316.81	59.58	422.58	67.75	50.56	1.311	1.340	1.178
Lu et al. [19]	GS-1	40.1	0.50	0.0049	29.94	474.00	51.85	579.38	56.72	754.79	64.55	57.5	0.902	1.123	0.986
	GS-2	45.7	0.64	0.0088	29.94	400.25	61.93	314.30	55.12	616.60	77.92	63.3	0.978	1.231	0.871
	GS-3	40.1	0.53	0.0049	29.94	488.23	50.98	579.11	55.20	839.43	66.79	56.37	0.904	1.185	0.979
	GS-4	45.7	0.67	0.0088	29.94	409.56	61.23	314.17	53.62	705.98	82.81	66.7	0.918	1.242	0.804
	GS-5	40.1	0.50	0.0049	29.94	474.00	51.85	579.38	56.72	754.79	64.55	53.79	0.964	1.200	1.054
	GS-6	45.7	0.64	0.0088	29.94	400.25	61.93	314.30	55.12	616.60	77.92	50.56	1.225	1.541	1.090
	BS-1	42.0	0.31	0.0022	29.94	649.19	43.63	783.72	46.57	858.49	48.18	52	0.839	0.927	0.896
	BS-2	42.0	0.40	0.0033	29.94	569.19	48.02	705.45	52.37	820.12	55.95	50.01	0.960	1.119	1.047
Kartal et al. [44]	B1S4	43.0	0.12	0.0011	31.28	574.15	60.82	895.33	65.03	287.95	57.01	69.6	0.874	0.819	0.934
	B2S3	43.0	0.26	0.0022	31.28	617.81	57.67	811.77	62.81	583.39	56.75	67.3	0.857	0.843	0.933
	B3S2	43.0	0.44	0.0033	31.28	653.36	55.33	728.14	58.34	878.82	64.31	66.7	0.830	0.964	0.875
	B4S1	43.0	0.68	0.0044	31.28	682.14	53.58	644.41	51.52	1174.26	79.02	70.2	0.763	1.126	0.734
	G1S4	35.0	0.22	0.0024	31.28	421.50	65.42	758.03	74.83	238.41	60.15	73.6	0.889	0.817	1.017
	G2S3	35.0	0.43	0.0048	31.28	419.39	65.65	585.67	74.94	483.74	69.29	73.9	0.888	0.938	1.014
	G3S2	35.0	0.63	0.0072	31.28	417.90	65.81	414.56	65.52	729.07	91.00	73.6	0.894	1.236	0.890
	G4S1	35.0	0.82	0.0096	31.28	416.80	65.93	244.59	45.58	974.39	121.48	72.5	0.909	1.676	0.629
Abbas et al. [25]	B1	83.8	0.33	0.0006	56.0	3295.57	86.01	1782.83	55.63	2567.46	71.46	40.3	2.134	1.773	1.380
	B2	63.5	0.53	0.0014	56.0	1937.78	106.40	1505.78	87.32	2160.42	116.14	74.2	1.434	1.565	1.177
	B3	83.8	0.67	0.0012	56.0	2487.31	110.12	1687.92	78.52	2666.74	117.12	65.3	1.686	1.794	1.202
	B4	83.8	0.47	0.0012	56.0	2286.52	118.49	1693.34	95.23	2449.71	124.82	82.3	1.440	1.517	1.157
	B5	83.8	0.40	0.0012	56.0	2266.05	120.37	1695.28	98.03	2301.24	121.73	88.9	1.354	1.369	1.103
	B6	83.8	0.50	0.0012	56.0	2374.32	116.14	1692.52	89.34	2465.09	119.67	71.8	1.618	1.667	1.244
	B7	83.8	0.39	0.0012	56.0	2151.87	127.82	1695.55	110.09	2253.32	131.74	82.9	1.542	1.589	1.328
Liu et al. [34]	2S6-2B6	55.0	0.50	0.0009	43.69	2074.47	46.74	1232.79	31.85	2110.50	47.37	39.94	1.170	1.186	0.798
	2S8-2B6	55.0	0.36	0.0009	43.69	1845.23	51.80	1235.60	41.14	1951.16	53.64	46.17	1.122	1.162	0.891
	2S10-2B6	55.0	0.26	0.0009	43.69	1621.95	57.92	1237.52	51.28	1746.31	60.06	59.57	0.972	1.008	0.861
	2S12-2B6	55.0	0.20	0.0009	43.69	1592.18	59.65	1238.82	53.56	1473.16	57.61	65.95	0.905	0.874	0.812
	2S6-2B8	55.0	0.64	0.0016	43.69	1576.39	58.98	1159.42	46.22	2066.68	73.67	52.84	1.116	1.394	0.875
	2S8-2B8	55.0	0.50	0.0016	43.69	1439.80	63.69	1162.07	55.28	1907.35	77.61	63.06	1.010	1.231	0.877
	2S10-2B8	55.0	0.39	0.0016	43.69	1404.07	65.84	1164.15	58.61	1679.73	74.06	72.93	0.903	1.015	0.804
Hussein et al. [26]	B15S60	48.0	0.18	0.0021	30.0	546.48	21.23	818.45	23.38	-6.40	16.66	19.16	1.108	0.869	1.220
	B25S61	48.0	0.18	0.0023	30.0	499.31	18.89	803.75	21.05	-108.77	14.34	18.66	1.012	0.769	1.128
	B15S45	48.0	0.18	0.0023	30.0	499.31	18.89	803.75	21.05	-108.77	14.34	18.67	1.012	0.768	1.128
Wang et al. [22]	B1.09-S0.25-0.23	51.0	0.81	0.0103	37.13	508.86	42.41	241.79	25.85	939.21	65.44	44.94	0.944	1.456	0.575
	B0.75-S0.36-0.48	51.0	0.68	0.0071	37.13	579.40	39.76	520.88	37.29	867.45	51.33	45.79	0.868	1.121	0.814
	B0.55-S0.53-0.96	51.0	0.51	0.0048	37.13	655.20	36.86	730.78	39.00	741.00	39.28	37.32	0.988	1.053	1.045
	B0.52-S0.52-1.0	51.0	0.50	0.0045	37.13	668.90	36.66	749.39	38.86	755.87	39.04	40.79	0.899	0.957	0.953
	B0.35-S0.54-1.56	51.0	0.39	0.0030	37.13	769.34	33.48	883.83	35.61	790.36	33.88	34.11	0.982	0.993	1.044
Liu et al. [18]	3S14-3RB14	55.5	0.50	0.0077	41.08	493.31	134.72	522.75	138.30	117.27	86.27	138.6	0.972	0.622	0.998
	3S12-3RB12	55.3	0.50	0.0056	41.08	649.74	112.66	725.15	119.70	649.30	112.61	125.9	0.895	0.894	0.951
	3S10-3RB10	55.4	0.50	0.0039	41.08	775.67	99.99	889.97	107.68	1080.58	120.23	114.4	0.874	1.051	0.941
Wei et al. [28]	H-A12-C55	50.1	0.50	0.0060	55.54	617.87	50.45	845.50	59.59	658.65	52.11	49.16	1.026	1.060	1.212
	H-B12-C55	55.6	0.50	0.0060	55.54	656.69	52.03	872.32	60.65	655.10	51.96	50.2	1.036	1.035	1.208
	H-C12-C55	124.2	0.50	0.0060	55.54	1019.30	66.36	1206.84	73.46	319.29	37.98	66.11	1.004	0.575	1.111
	H-G10-C55	48.66	0.41	0.0041	55.54	700.53	45.03	1053.49	55.06	751.63	46.50	44.51	1.012	1.045	1.237
	H-G12-C55	45.04	0.50	0.0060	55.54	579.99	48.90	820.83	58.61	658.86	52.12	48.04	1.018	1.085	1.220
	H-G14-C55	43.42	0.58	0.0081	55.54	499.78	52.99	567.52	56.69	565.31	56.57	50.45	1.050	1.121	1.124
	H-G16-C55	41.08	0.64	0.0106	55.54	431.40	56.47	281.44	45.60	471.83	59.32	52.14	1.083	1.138	0.875
	H-G12-C30	45.04	0.50	0.0060	33.94	466.60	42.32	567.27	46.10	528.78	44.67	39.22	1.079	1.139	1.175
	H-G12-C40	45.04	0.50	0.0060	41.96	521.61	45.43	661.46	50.86	577.10	47.60	45.47	0.999	1.047	1.118
	H-G12-C50	45.04	0.50	0.0060	51.05	566.23	48.02	768.08	56.07	631.80	50.66	47.37	1.014	1.070	1.184
Mean value													1.035	1.103	1.006
Standard deviation													0.223	0.313	0.163

## Data Availability

The raw data supporting the conclusions of this article will be made available by the authors on request.
